# Markers for the central serotonin system correlate to verbal ability and paralinguistic social voice processing in autism spectrum disorder

**DOI:** 10.1038/s41598-020-71254-w

**Published:** 2020-09-03

**Authors:** Yuko Yoshimura, Mitsuru Kikuchi, Daisuke N. Saito, Tetsu Hirosawa, Tetsuya Takahashi, Toshio Munesue, Hirotaka Kosaka, Nobushige Naito, Yasuomi Ouchi, Yoshio Minabe

**Affiliations:** 1 Institute of Human and Social Sciences, Kanazawa University, Kakuma-machi, Kanazawa, 920-1192 Japan; 2 Research Center for Child Mental Development, Kanazawa University, 13-1 Takara-machi, Kanazawa, 920-8640 Japan; 3 Department of Psychiatry and Neurobiology, Graduate School of Medical Science, Kanazawa University, 13-1 Takara-machi, Kanazawa, 920-8641 Japan; 4 Department of Neuropsychiatry, University of Fukui, Matsuoka-shimoaizuki, Eiheiji-cho, Yoshida-gun, 910-1193 Japan; 5 Research Center for Child Mental Development, University of Fukui, 23-3 Matsuoka-shimoaizuki, Eiheiji-cho, Yoshida-gun, Fukui 910-1193 Japan; 6 Department of Biofunctional Imaging, Medical Photonics Research Center, Hamamatsu University School of Medicine, 1-20-1 Handayama, Higashi-ku, Hamamatsu, 431-3192 Japan

**Keywords:** Autism spectrum disorders, Magnetoencephalography

## Abstract

Impairment in verbal communication abilities has been reported in autism spectrum disorder (ASD). Dysfunction of the serotonergic system has also been reported in ASD. However, it is still unknown how the brain serotonergic system relates to impairment in verbal communication abilities in individuals with ASD. In the present study, we investigated the correlation between brain serotonergic condition and brain sensitivity to paralinguistic stimuli (i.e., amplitude in the human voice prosodic change-evoked mismatch field) measured by magnetoencephalography (MEG) or verbal ability in 10 adults with ASD. To estimate the brain serotonergic condition, we measured the serotonin transporter nondisplaceable binding potential cerebrum-wide using positron emission tomography with [11C]N,N-dimethyl-2-(2-amino-4-cyanophenylthio)benzylamine ([11C] DASB). The results demonstrated a significant positive correlation between brain activity to paralinguistic stimuli and brain serotonin transporter binding potential in the left lingual gyrus, left fusiform gyrus and left calcarine cortex. In addition, there were significant positive correlations between verbal ability and serotonergic condition in the right anterior insula, right putamen and right central operculum. These results suggested that the occipital cortex is implicated in recognition of the prosodic change in ASD, whereas the right insula-involved serotonergic system is important in nurturing verbal function in ASD.

**Trial registration**: UMIN000011077.

## Introduction

Autism spectrum disorder (ASD), which is characterized by impaired social cognition and communication and repetitive and/or obsessive behaviour and interests^1^, is an aetiologically and symptomatologically complicated disorder of brain development. Regarding the baseline idea of social impairment in ASD, deficits in verbal communication^2,3^ from early childhood are thought to prevent the normal acquisition of social ability. Biological mechanisms related to impairment in verbal communication in ASD should be clarified to promote an objective understanding of pathology in ASD.

A variety of human and animal studies support the hypothesis that dysfunction in the serotonergic system is a contributing factor in the development of ASD^4–7^. Due to the pleiotropic role of the serotonergic system across multiple brain systems during development, this system seems to be a logical candidate to explain the diversity in ASD symptoms. Notably, many intriguing studies have reported the relationship between communication ability and serotonin. Genetically, variants of genes in the serotonin transporter (SERT) contribute to various cognitive impairments (e.g., delayed language onset and intellectual disability) in individuals with ASD^8,9^. Pharmacologically, selective serotonin reuptake inhibitors (SSRIs) have been used to counteract various symptoms of the disorder^10^, and an open-label trial of fluoxetine (an SSRI) demonstrated that responders among children with ASD showed marked increases in language acquisition compared with their pretreatment status^11^. Regarding brain imaging studies with positron emission tomography (PET), two previous studies have demonstrated significant reductions in cerebral serotonin synthesis capacity in children with ASD^12,13^ and demonstrated that abnormally asymmetric development of the brain serotonergic system could affect language development in children with ASD^13^. In adults with ASD, PET studies have also demonstrated decreases in the level of SERT binding potential (BP_ND_) throughout the brain, with SERT BP_ND_ reduction in the anterior and posterior cingulate cortices being linked to impairment of social cognition^6^. Although the sample size is small (*n* = 8), an earlier study also found trends of decreased SERT BP_ND_ in amygdala, insula, thalamus uncus, and midbrain in individuals with Asperger’s Disorder^14^.

Auditory mismatch negativity (MMN) is a neural signature of a preattentive index of auditory discrimination that is traditionally elicited in an oddball paradigm^15^. In the oddball paradigm, presentations of sequences of repetitive stimuli are infrequently interrupted by a deviant stimulus. The MMN is elicited by sounds that deviate from some regular property of the preceding sounds and are affected in a large number of different clinical conditions such as schizophrenia^16^, bipolar disorder^17^, stroke and aphasia^18,19^, epilepsy^20^, Parkinson’s disease^21^, dementia^22^, Alzheimer’s disease^23^, dyslexia^24^ and ASD^25^. The magnetic mismatch field (MMF), the neuromagnetic component of the MMN, is quantified from the average deviant waveform by subtracting the average waveform generated by the brain in response to standard stimuli. MMN/MMF typically peaks at 100–250 ms from the onset of the stimulus change^15,26^. In healthy populations, the amplitude and latency of MMN/MMF is considered an indicator of change detection and has been used to probe speech discrimination^27,28^. In individuals with ASD, a number of studies have reported atypical MMN/MMF^29–35^ and MMN/MMF is thought to be an indicator that physiologically explains core symptoms^31^, language impairment/delay^32,34^ and preference for human voices^33^. Individuals with ASD showed a reduced MMF amplitude response in the left hemisphere compared to that of control subjects^34^. Additionally, it has been reported that MMF latency is significantly prolonged in children with ASD compared with control subjects. Furthermore, this delay was most pronounced in individuals with language impairment^32^. Intriguingly, using acute tryptophan depletion, some previous studies in healthy subjects demonstrated that MMN/MMF could be affected by malfunction of the serotonergic system ^36,37^.

Although it is still unknown how the brain serotonergic system relates to the verbal intelligence quotient (VIQ) and/or the MMF evoked by paralinguistic but socially communicative human vocalizations in adults with ASD, deviations in these measurements (i.e., brain serotonergic system^6,12,13^, VIQ^38^ and MMF^29–35^) have been reported in previous studies on ASD. In the present study, we hypothesized that the function of the serotonergic system is related to verbal ability and/or the brain response evoked by paralinguistic prosodic voice change in ASD. To test this hypothesis, we investigated whether the decrease in brain SERT is related to the low language ability and/or decrease in MMF amplitude to prosodic change in individuals with ASD. Specifically, we used PET to measure whole-brain SERT BP_ND_ in 10 adults with ASD, and we investigated correlation between SERT BP_ND_ and VIQ or MMF in response to paralinguistic and socially communicative human utterances using magnetoencephalography (MEG). Furthermore, as a point of comparison for VIQ, we used the performance intelligence quotient (PIQ) component of the WAIS-R or WAIS-III: a component that measures non-linguistic perceptual organization and processing speed.

## Results

To investigate correlation with whole-brain SERT BP_ND_ using PET, we used mismatch responses in the superior temporal area in the right and left hemispheres as regions of interest (ROIs). We selected the superior temporal lobe and the banks of the superior temporal sulcus as ROIs based on the prediction that MMF generators would be located primarily in the superior temporal regions^15,39–42^.

**Figure 1:**
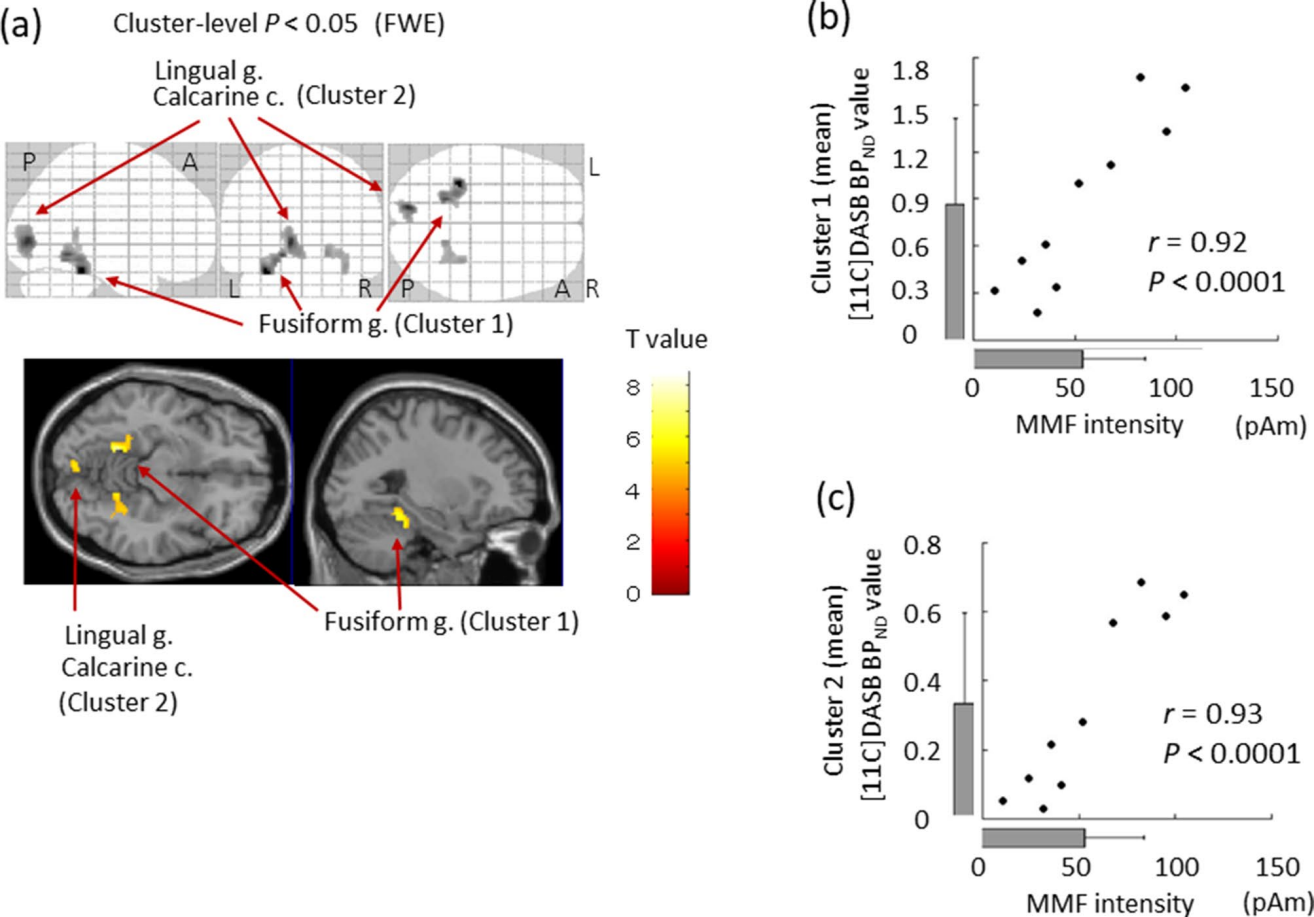
(**a**) Voxel-wise image analyses of [11C] DASB BP_ND_ performed using SPM software (SPM12; Wellcome Department of Cognitive Neurology, Institute of Neurology, London, England, https://www.fil.ion.ucl.ac.uk/spm/software/). SPM analyses with multiple regression models in which the MMF intensity evoked in the right superior temporal area was used as an independent variable for the binding of SERT in the brain. Significant positive correlation was found for the left lingual gyrus, left fusiform gyrus and left calcarine cortex. Scatterplot showing the correlation between the [11C] DASB BPND mean value in the (**b**) Cluster 1 or (**c**) Cluster 2 and MMF intensity evoked in the right superior temporal area. The bar graphs show the average value. Error bars correspond to one standard deviation. *L* left hemisphere, *R* right hemisphere, *A* anterior, *P* posterior, *G* gyrus, *C* cortex, *SERT* serotonin transporter, *Cluster 1* significant voxels in fusiform gyrus, *Cluster 2* significant voxels in lingual gyrus and calcarine cortex.

In the left lingual gyrus *(r* = 0.94, *P* < 0.001, uncorrected for the peak voxel), left fusiform gyrus (*r* = 0.95, *P* < 0.001, uncorrected for the peak voxel) and left calcarine cortex (r = 0.95, *P* < 0.001, uncorrected for the peak voxel) (*P* < 0.05, family wise error corrected for cluster size with a height threshold of *P* < 0.001) (Table 1, Fig. 1), statistical parametric mapping (SPM) demonstrated that SERT BP_ND_ are positively correlated with MMF intensity evoked in the right superior temporal area. Examination of each cluster level specifically indicates that the mean SERT BP_ND_ in Cluster 1 (the left lingual and fusiform gyrus: 257 voxels) is positively correlated with the MMF intensity (*r* = 0.92, *P* < 0.0001, uncorrected). Also, the mean SERT BP_ND_ in Cluster 2 (the left calcarine cortex and lingual gyrus: 273 voxels) is positively correlated with the MMF intensity (*r* = 0.93, *P* < 0.0001, uncorrected). To evaluate the existence of a possible mood and anxiety effect on the significant relationship found for these clusters, we used multiple linear regression to predict the mean SERT BPND in Clusters 1 and 2 (i.e., dependent variable) using MMF intensity, depression (Zung Self-rating Depression Scale (SDS)), and anxiety (State-Trait Anxiety Inventory (STAI)-Trait) scores as predictors. For these additional analyses, significance was inferred for *P* < 0.001. In the multiple regression model for the Cluster 1, MMF intensity was the significant predictor of the mean SERT BPND (β = 0.93, *P* < 0.001), although statistical significance was not found for depression (β = -0.20, *P* > 0.05) or anxiety (β = 0.23, *P* > 0.05) scores. From the multiple regression model for Cluster 2, MMF intensity was found to be a significant predictor of the mean SERT BPND (β = 0.94, *P* < 0.001), although statistical significance was not found for depression (β = -0.17, *P* > 0.05) or anxiety (β = 0.18, *P* > 0.05) scores.

**Table 1:** Cortical regions where a significant positive correlation was found between SERT BP_ND_ and MMF intensity in the right hemisphere.

Cluster level			Peak level				MNI co-ordinates			Side	Location
*P* (FWE)	Size (voxels)	*r*	*t*	z	*P* (uncorrected)	*r*	x	y	z	L/R	
0.018*	Cluster 1	0.92	8.47	4.18	< 0.001	0.95	− 32	− 40	− 22	L	Fusiform g
	257		7.37	3.95	< 0.001	0.94	− 18	− 52	− 10	L	Lingual g
			6.64	3.77	< 0.001	0.92	− 24	− 42	− 14	L	Fusiform g
0.014*	Cluster 2	0.93	7.88	4.06	< 0.001	0.95	− 12	− 86	0	L	Calcarine c
	273		5.69	3.50	< 0.001	0.90	− 4	− 86	− 8	L	Lingual g
0.066	181	0.90	5.65	3.49	< 0.001	0.90	18	− 52	− 8	R	Lingual g
			5.46	3.43	< 0.001	0.89	34	− 38	− 20	R	Fusiform g
			5.03	3.29	0.001	0.87	28	− 48	− 8	R	Lingual g

Statistical significance was inferred for a cluster-level *P* < 0.05, FEW-corrected, and for a voxel-level *P* < 0.001, uncorrected. MNI brain atlas co-ordinates: x = distance in millimetres to the right (+) or the left (–) side of the midline; y = distance anterior (+) or posterior (–) to the anterior commissure; and z = distance superior (+) or inferior (–) to a horizontal plane through the anterior and posterior commissures. MMF, mismatch field. *, *P* < 0.05. FWE, family-wise error. *r*, correlation coefficient (effect size). R, right. L, left. G, gyrus. C, cortex.

There was no significant relationship between brain SERT BPND and MMF intensity evoked in the left superior temporal area.

**Figure 2:**
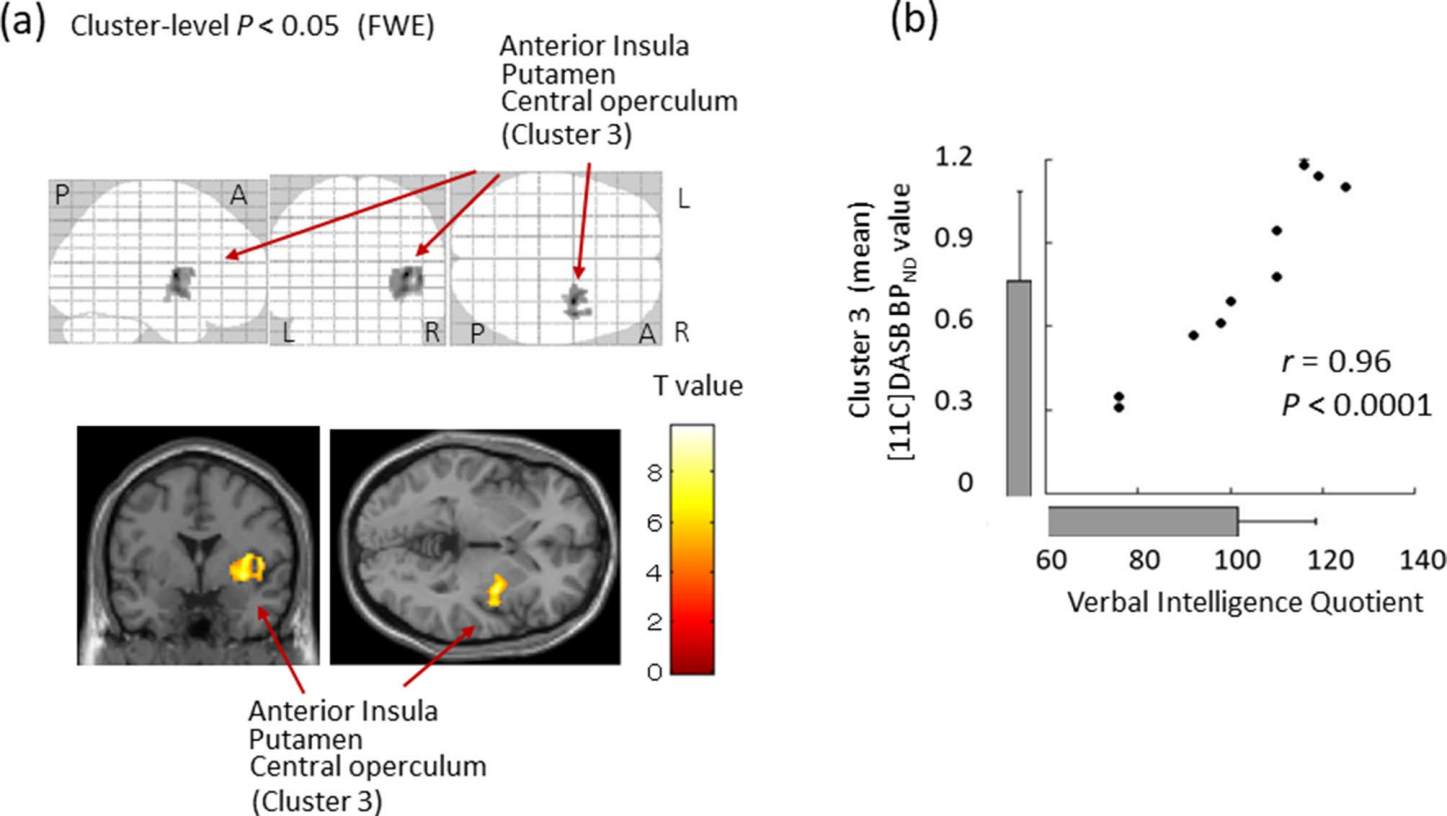
(**a**) Voxel-wise image analyses of [11C] DASB BP_ND_ performed using SPM software (SPM12; Wellcome Department of Cognitive Neurology, Institute of Neurology, London, England, https://www.fil.ion.ucl.ac.uk/spm/software/). SPM analyses with multiple regression models in which verbal IQ was used as an independent variable for the binding of brain SERT. Significant positive correlation was found for the right anterior insula, right putamen and right central operculum. (**b**) Scatterplot showing the correlation between the [11C] DASB BPND value (mean) in the Cluster 3 and MMF intensity evoked in the right superior temporal area. The bar graphs show average values. Error bars correspond to one standard deviation. *L* left hemisphere, *R* right hemisphere, *A* anterior, *P* posterior, *SERT* serotonin transporter. *Cluster 3* significant voxels in anterior insula, putamen and central operculum.

In the right anterior insula (*r* = 0.97, *P* < 0.001, uncorrected for the peak voxel), right putamen (*r* = 0.95, *P* < 0.001, uncorrected for the peak voxel) and right central operculum (*r* = 0.95, *P* < 0.001, uncorrected for the peak voxel) (*P* < 0.05, family wise error corrected for cluster size with a height threshold of *P* < 0.001) (Table 2, Fig. 2), the SPM results demonstrated that SERT BP_ND_ is positively correlated with VIQ, although no significant correlation was found with PIQ. Specific examination of the cluster level revealed the mean SERT BP_ND_ in Cluster 3 (the right anterior insula, putamen and central operculum: 450 voxels) as positively correlated with MMF intensity (*r* = 0.96, *P* < 0.0001, uncorrected). To evaluate the existence of a possible mood and anxiety effects on the significant relation found for this cluster, we used multiple linear regression to predict the mean SERT BP_ND_ in Cluster 3 (i.e., dependent variable) using VIQ, depression (SDS) and anxiety (STAI-Trait) scores as predictors. For this additional analysis, significance was inferred for *P* < 0.001. In the multiple regression model, VIQ was found to be a significant predictor of the mean SERT BP_ND_ (β = 0.95, *P* < 0.001), but no significance was found for depression (β = -0.16, *P* > 0.05) or anxiety (β = 0.19, *P* > 0.05) scores.

**Table 2:** Cortical regions for which significant positive correlation was found between the serotonin transporter binding potential and verbal IQ.

Cluster-level			Peak-level				MNI co-ordinate			Side	Location
*P* (FWE)	Size (voxels)	*r*	*t*	z	*P* (uncorrected)	*r*	x	y	z	L/R	
0.001*	Cluster 3	0.96	9.92	4.44	< 0.001	0.97	36	− 2	8	R	Anterior insula
	450		8.57	4.20	< 0.001	0.95	34	0	− 4	R	Putamen
			8.49	4.19	< 0.001	0.95	46	0	6	R	Central operculum

Statistical significance was inferred at a cluster-level *P* < 0.05, FWE corrected, and a voxel-level *P* < 0.001, uncorrected. *r*, correlation coefficient (effect size).

## Discussion

In this study, we investigated brain serotonergic systems using PET and examined brain sensitivity to human paralinguistic voice stimuli using MMF in adults with ASD. Our results showed that increased voice-evoked MMF in the right superior temporal area coincided with more SERT BP_ND_ in the left lingual gyrus, left fusiform gyrus, and left calcarine cortex. Intriguingly, several previous studies demonstrated a diminished MMN/MMF amplitude for emotional voice perception predominantly in the right hemisphere in individuals with ASD^43,44^. Charpentier et al.^44^ reported that MMN for emotional deviancy was less lateralized to the right hemisphere in children with ASD than in control children. Kujala et al.^43^ also reported that comparisons of MMN amplitude for a deviant stimulus with a scornful emotional connotation revealed a significant group difference between individuals with Asperger syndrome and control subjects. These previous studies demonstrated that right hemispheric dominant dysfunction is associated with ASD using human voice stimuli. However, it remains unclear how the serotonin system was involved in these right-brain dominant dysfunctions. The present study is the first to suggest that the lower MMF intensity evoked in the right superior temporal area reflects malfunction of the serotonergic system in the bilateral visual association areas in individuals with ASD. It seems strange that the function of auditory human voice processing is related to the visual association cortices. However, some previous studies have also reported the role of visual association cortices in paralinguistic information processing. For instance, not only during visual stimuli (e.g., emotional faces)^45^ but also during voice stimulation^46^, activations in these occipital cortices were reported. A previous functional magnetic resonance imaging (fMRI) study also reported that a mother’s voice increased her child’s brain activity not only in the auditory area but also in the sub-region of the fusiform gyrus associated with face perception^47^. Furthermore, it was reported that individuals with higher ASD traits have dysfunction in these occipital cortices during emotional human voice processing. Blasi et al.^48^ using fMRI, demonstrated that infants at low familial risk for ASD showed stronger sensitivity to sad vocalizations in the fusiform gyrus than did infants at high familial risk for ASD. These studies suggested that the visual cortex has an important role in the neural processing of human social information (e.g., prosody) in both typically developed individuals and those with familial risk for ASD, and our results suggested that dysfunction of the serotonergic system in these areas resulted in lower paralinguistic brain sensitivity in individuals with ASD. However, it is unclear why cross-hemispheric correlation (i.e., right hemispheric brain response and left hemispheric serotonin system) was stronger than intra-hemispheric correlation (i.e., right hemispheric brain response and right hemispheric serotonergic system) in the present study. To draw definitive conclusion, further study with larger sample size is necessary.

In this study, we also investigated the relationship between the brain serotonergic system and VIQ in adults with ASD. Our results showed a significant positive correlation between VIQ and the level of SERT BP_ND_ in the right hemisphere (i.e., right anterior insula, right putamen and right central operculum). In children with ASD, one previous PET study investigated brain serotonin synthesis with the tracer alpha-[11C]-methyl-L-tryptophan (AMT)^13^. Contrary to our results, the authors reported that children with ASD having decreased cortical AMT binding in the left hemisphere manifested a higher prevalence of severe language impairment^13^. The different laterality of the brain serotonergic system highlighted in our and their studies might be due to the differences in age/sex, intellectual levels (i.e., adults without language impairment vs. children with language impairment), PET tracers and methods applied to adults and children. Differences in the developmental trajectory of serotonin synthesis between ASD and typically developing (TD)^12^ might also contribute to these inconsistent results. Although the matter remains open to speculation, there is a possibility that the enhanced activity of the serotonergic system in the right hemisphere acted as a compensatory mechanism to support verbal ability in adult participants with ASD.

The insula shares reciprocal functional and structural connections with linguistic, motor, orbital cortex, frontal operculum and sensory brain areas^49^. The insula is also involved in auditory perception, speech processing, language memory and the comprehension of syntactically complex sentences^50–53^. Recently, many researchers have suggested a link between atypical activation and connectivity of the insular cortex in ASD^54–56^. Actually, ASD has been linked with dysfunction not only in the insula but also in the basal ganglia in both their motor and non-motor domains^57^. The right anterior putamen has been reported to contribute to speech development^58^. Therefore, our current results suggest that a network including the right insula, right putamen and right central operculum plays an important role in verbal ability along with other social abilities in adults with ASD.

The present study has some limitations. A major limitation is the lack of a control group. Normal values of SERT BP from older participants were chosen as counterparts for Alzheimer's patients, as reported from an earlier study^59^. The BPND values in the prefrontal and temporal cortices found in the present study were, respectively, 0.35 ± 0.18 and 0.4 ± 0.11, which suggests that the range of the DASB BPND values estimated in our ASD participants is consistent with the range of normal young counterparts. However, it is unclear whether the ASD participants had abnormal MMF or SERT BP or whether the observed correlations were specific for the subjects with ASD. Second, the other major limitation of the present study is the small sample size. Therefore, we must consider the following possibilities: low probability of finding a true effect, low positive predictive value, and an exaggerated estimate of the magnitude of the effect when a true effect is discovered. It will be important to replicate these findings using a larger sample that includes both sexes and a broadened age range. Third, because of our study design (i.e., PET was conducted at rest and not during auditory social stimulation), we cannot draw any conclusions regarding causal links between the serotonin system and auditory social information. Fourth, the 40 stimulus trials in this study represent a size that is less than typical for an MMN/F study. The averaged data for a sufficient number of trials must be considered.

In conclusion, the present study suggested that the serotonergic system in the left occipital cortex is important in recognition of the paralinguistic prosodic change in ASD and that the serotonergic system in the right insula-striatum-operculum region is implicated in nurturing verbal ability in adults with ASD. Whereas dysfunction of the central serotonin system has been reported in individuals with ASD, we have now demonstrated that this deviation in some brain regions is associated with deficits in language ability and paralinguistic social voice processing.

## Methods

### Participants

Ten adult men with ASD (mean [SD] age, 30.3 [5.8] years; age range, 23–41 years) participated in the experiment. All subjects were right-handed native Japanese individuals with an intelligence quotient (IQ) greater than 70 (mean [SD] IQ, 102.2 [13.0]; IQ range, 85–130) as measured using the Wechsler Adult Intelligence Scale-Revised (WAIS-R)^60^ or the Wechsler Adult Intelligence Scale Third Edition (WAIS-III)^61^ (Table 3). The diagnosis of ASD was based on the DSM-5^1^ and the Autism Diagnostic Observation Schedule–Generic (ADOS-G)^62^. All participants were screened using a Structured Clinical Interview for DSM-IV-TR diagnosis to exclude co-morbid psychiatric illness (e.g., intellectual disability) other than history of insomnia disorder or major depressive disorder. Individuals with a history of neurological disorder (e.g., epilepsy, head injury) were also excluded. The mean ADOS score (range) of the included participants was 13.3 (9–18). We used the VIQ component of the WAIS-R (one subject) or the WAIS-III (nine subjects) as an index of verbal ability. As a point of comparison for VIQ, we employed the performance intelligence quotient (PIQ) component of the WAIS-R or WAIS-III, a component that measures non-linguistic perceptual organization and processing speed. We also assessed anxiety and depressive symptoms using the STAI^63^ and SDS^64^, respectively, because those symptoms can be associated with the serotonergic system^65,66^.

**Table 3:** Clinical characteristics.

	Mean (SD)	Range
Age, yr	30.3 (5.8)	23–41
Education, yr	14.3 (1.9)	12–16
ADOS score	13.3 (2.5)	9–18
SDS	49.5 (8.6)	34–61
STAI-trait	57.9 (9.6)	42–69

*ADOS* autism diagnostic observation schedule, *SDS* Zung self-rating depression scale, *STAI* state-trait anxiety inventory.

We added no restriction on clinical treatment in this study. Seven of the included patients were naïve to medical treatment for at least 6 months before the experiment. For three patients, medication including SSRI (1 patient) and benzodiazepines (3 patients) was applied continuously. The drug type and quantity were not changed for these patients for 4 weeks before measurements. Written informed consent was obtained from all participants with ASD prior to enrolment. The Ethics Committee of Kanazawa University Hospital and Hamamatsu University School of Medicine approved the methods and procedures used in this study, which was performed in accordance with the Declaration of Helsinki. The study was registered with the University Hospital Medical Information Network Clinical Trials Registry (number UMIN000011077). The participants were the same as those of a previously reported study^67^. Therefore, these experiments were conducted as an open-label, single-arm, non-randomized, and non-controlled study. First clinical assessment data, MEG and PET scan data (i.e. baseline data for an earlier cited study)^67^ were used for the present study.

### MEG measurement and data analysis for MMF

The method of MMF analysis is based on our previous research^34^. Magnetic fields were measured using a whole-head MEG system for adults in a magnetically shielded room (Daido Steel, Nagoya, Japan) in the MEG Center of Ricoh Company, Ltd., in Japan. This system (MEG vision PQA160C; Yokogawa Electric Corporation, Kanazawa, Japan) employs 160 channels of axial gradiometers, where the coil diameter of the sensors is 15.5 mm and the baseline is 50.0 mm. Band-pass-filtered MEG data (0.16–200 Hz) were collected at a sampling rate of 2000 Hz. Structural magnetic resonance imaging (MRI) scans were acquired using a 1.5 T MRI scanner (GE Yokogawa) with a T1-weighted Fast SPGR sequence and the following parameters: repetition time = 8.364 ms, echo time = 3.424 ms, flip angle = 12°, field of view = 260 mm, matrix size = 512 × 512 pixels, slice thickness = 1 mm, and 176 transaxial images. All subjects underwent T1-weighted MRI with spherical lipid markers placed at 5 MEG fiducial points to facilitate the superimposition of the MEG coordinate system on that of MRI.

We used typical oddball sequences consisting of standard stimuli (230 times, 83%) and deviant stimuli (45 times, 17%). We used the Japanese syllable “ne” because this syllable is a sentence-ending particle in Japanese and conveys prosodic information^68^. The syllable “ne” expresses a speaker’s request for acknowledgement or empathy from the listener^69^. This syllable can be pronounced in two different ways. A repetitive series of utterances of “ne” pronounced with a flat tone (/ne/) was used as the standard. This stimulus carries no intonational information. As a deviant stimulus, we used “ne” pronounced with a high falling tone (/Ne/), which carries intonational information that gives the listener a feeling of “being spoken to”^70,71^. The duration of standard stimulus is 342 ms (consonant /n/ was 65 ms duration and vowel /e/ was 227 ms duration). The duration of deviant stimulus is 341 ms (consonant /N/ was 53 ms and vowel /e/ was 288 ms). The interstimulus interval (ISI) was 818 ms.

The continuous MEG data were subsequently epoched into 100-ms pre-stimulus intervals and 900-ms post-stimulus intervals. The 50-ms pre-stimulus interval (i.e., -50 to 0 ms) was used for the baseline correction. Epochs contaminated by muscle, heartbeat or eye-blink artefacts that included field amplitudes greater than ± 4 pT were excluded from analyses. Brainstorm software^72^, which is documented and freely available for download online under the GNU general public license (https://neuroimage.usc.edu/brainstorm), was used for subsequent analyses. Typical eye blinks and heartbeats were manually identified in raw data for each participant and were corrected if they were found to be artefactual. The trials of each type of stimulus were subsequently averaged after baseline correction (− 50 to 0 ms). MMF signals were obtained with an average time window of 100–250 ms^42^. The mean number of trials for each stimulus (i.e., rare and frequent) was 40 ± 6 (mean ± standard deviation). The MMF responses were calculated by subtracting the average response to the standard stimuli (flat tone /ne/) from the average response to the deviant stimuli (falling tone /Ne/).

We estimated the signal source of the MMF and the individually estimated anatomical locations for the participants. The anatomical locations of the activating regions were based on the Desikan–Killiany gyrus atlas provided by FreeSurfer (open-source software: https://surfer.nmr.mgh.harvard.edu/)^73^. Source reconstruction was performed using Brainstorm^72^. To estimate the brain sources, we used an MEG approach that placed an anatomical constraint on the estimated sources by assuming that the recorded brain activity of each lay in the cortical mantle^74^. The inverse solution was calculated for each using Tikhonov-regularized minimum-norm estimates^75^ with a depth-weighted minimum-norm estimator (wMMN)^76^. We set the depth–weight parameter to 0.5 ^77^. To investigate the correlations with cerebrum-wide SERT BP_ND_, we used mismatch responses in the superior temporal area in the right and left hemispheres as ROIs. We selected the superior temporal lobe and the banks of the superior temporal sulcus as ROIs based on the prediction that MMF generators would be located primarily in the superior temporal regions^15,39–42^.

### SERT imaging procedure and data analysis for PET

To measure the non-displaceable binding potential (BP_ND_) of SERT, PET was performed as described previously^78–80^ using high-resolution brain SHR12000 tomography (Hamamatsu Photonics K.K.) with an intrinsic resolution of 2.9 × 2.9 × 3.4 mm at full width at half maximum, 38 slices, and a 163-mm axial field of view. After head fixation using a thermoplastic face mask and a 10-min transmission scan for attenuation correction, serial scanning (4 × 30 s, 20 × 60 s, and 14 × 300 s) was performed during a period of 92 min after a bolus injection (taking 1 min) of a 300-MBq dose of [11C]N,N-dimethyl-2-(2-amino-4-cyanophenylthio)benzylamine ([11C] DASB) with specific activity of more than 90 GBq/μmol. PET examinations were conducted one week after MEG measurements.

[11C] DASB is a highly selective radioligand that binds reversibly to SERT with high affinity^81–84^. [11C] DASB enables non-invasive estimates of the SERT BP^85^, which has been shown using a reference tissue method to correlate well with SERT density^82,84^. The MRI measurement and a mobile PET gantry allowed us to reconstruct PET images parallel to the intercommissural line without re-slicing. Using this approach, we allocated the original PET images to the brain structural images^78,79^. The MRI and PET examinations were performed without sedation. After we estimated the BP of [11C] DASB based on a multilinear reference tissue model^79^, we constructed parametric images for all participants using biomedical imaging software (PMOD, ver. 2.5; PMOD Technologies Ltd., Zurich, Switzerland). Subsequent voxel-wise image analyses of [11C] DASB BP_ND_ were performed using SPM software (SPM12; Wellcome Department of Cognitive Neurology, Institute of Neurology, London, England).

### Statistical analysis

Correlation analyses were performed between the MMF intensity in the right or left hemisphere and the SERT BP_ND_ cerebrum-wide. In addition, correlation analyses were performed between VIQ or PIQ and the SERT BP_ND_ cerebrum-wide. For SPM analysis, voxel-based correlations were computed using a parametric multiple regression model for which *P* = 0.05 for family wise error corrected for cluster size (height threshold of *P* < 0.001).

### Ethics approval and consent to participate

The Ethics Committee of Kanazawa University Hospital approved the methods and procedures, which were performed in accordance with the Declaration of Helsinki.

### Consent for publication

Written informed consent was obtained from all participants before enrolment.

## Data Availability

The datasets collected during or analysed during the current study are available from the corresponding author upon reasonable request.

## Abbreviations

ASD: Autism spectrum disorder
SERT: Serotonin transporter
SSRIs: Selective serotonin reuptake inhibitors
PET: Positron emission tomography
BP_ND_: Binding potential
MMN: Mismatch negativity
MMF: Mismatch field
VIQ: Verbal intelligence quotient
MEG: Magnetoencephalography
WAIS-R: Wechsler adult intelligence scale-revised
WAIS-III: Wechsler adult intelligence scale third edition
ADOS-G: Autism diagnostic observation schedule-generic
PIQ: Performance intelligence quotient
MRI: Magnetic resonance imaging
fMRI: Functional magnetic resonance imaging
ROIs: Regions of interest
[11C] DASB: [11C]N,N-dimethyl-2-(2-amino-4-cyanophenylthio)benzylamine
SPM: Statistical parametric mapping
SDS: Zung self-rating depression scale
STAI: State-trait anxiety inventory
AMT: Alpha-[11C]-methyl-L-tryptophan
TD: Typically developing
